# The effect of reward value on the performance of long-tailed macaques (*Macaca fascicularis*) in a delay-of-gratification exchange task

**DOI:** 10.5194/pb-11-19-2024

**Published:** 2024-07-26

**Authors:** Judit J. Stolla, Stefanie Keupp

**Affiliations:** 1 Cognitive Ethology Laboratory, German Primate Center – Leibniz Institute for Primate Research, Kellnerweg 4, 37077 Göttingen, Germany; 2 Department for Primate Cognition, Johann-Friedrich-Blumenbach Institute, Georg-August-Universität Göttingen, Kellnerweg 4, 37077 Göttingen, Germany; 3 Leibniz ScienceCampus, German Primate Center – Leibniz Institute for Primate Research, Kellnerweg 4, 37077 Göttingen, Germany

## Abstract

In the context of a global research initiative called ManyPrimates, scientists from around the world collaborated to collect data aimed at comparing the ability of various primate species to delay gratification. Our contribution to this project involved collecting data from long-tailed macaques (*Macaca fascicularis*). Our findings indicated that these macaques rarely opted to exchange a given food item for a larger food reward at a later time. However, we suspected that the experimental protocol might not accurately capture the macaques' actual capacity to delay gratification. Specifically, possessing a highly desirable food item might discourage the monkeys' participation in food exchange and delay-of-gratification tasks. To explore whether this potential mental distraction was affecting their performance, we conducted experiments on six long-tailed macaques under two different conditions. In these conditions, we examined how the value of the exchange item influenced the frequency of exchanges. In one condition, subjects received a high-value food item, while in the other, they received a low-value food item, both of which could be exchanged for three high-value food items. When we reduced the value of the exchange item, the long-tailed macaques displayed significantly improved abilities to delay gratification within the exchange task. These findings strongly suggest that the possession of a high-value item significantly contributed to the low performance of these monkeys in the original delay-of-gratification exchange protocol and raise the question of which performance reflects the monkeys' underlying delay-of-gratification competence more adequately.

## 1 Introduction

The capacity to resist the allure of immediate gratification in favour of securing a more valuable reward in the future is a critical skill when it comes to future-oriented decisions (Pelé et al., 2011). Commonly referred to as “delay of gratification”, this cognitive trait has been observed across a wide range of species, including chimpanzees (Beran and Evans, 2006; Evans and Beran, 2007a), bonobos (Stevens et al., 2011), capuchin monkeys (Addessi et al., 2013; Bramlett et al., 2012), squirrel monkeys (Anderson et al., 2010), rhesus macaques (Evans and Beran, 2007b), long-tailed macaques (Pelé et al., 2010), mice (Gao et al., 2021), various parrot species (Brucks et al., 2022; Pepperberg and Rosenberger, 2022; Vick et al., 2010), dogs and wolves (Range et al., 2020), and diverse fish species (Aellen et al., 2021; Schnell et al., 2021).

The investigation of delay-of-gratification ability has involved a diverse range of experimental methods and designs. Broadly, these approaches can be categorized into two main types – sustained choice tasks and commitment choice tasks – each offering unique insights into the subject's capacity to delay gratification (Reynolds et al., 2002). Sustained choice tasks involve scenarios where subjects must maintain their selected option throughout a designated delay period. In these tasks, the choices are not final until the subject decides to act. Examples of commonly used sustained choice tasks are the accumulation task and the exchange task. In the accumulation task, subjects are presented with a growing pile of food within their reach. The trial concludes as soon as the subject decides to consume the reward (Anderson et al., 2010; Beran and Parrish, 2021). In exchange tasks, subjects initially receive a smaller food item which they can trade for a larger reward at the end of a specified delay period (Beran et al., 2016; Reynolds and Schiffbauer, 2005). In contrast, commitment choice tasks present situations where a choice is irreversible once a decision is made (Reynolds et al., 2002). For instance, the rotating food tray task presents subjects with two differently sized food rewards on a moving tray, and subjects must refrain from selecting the smaller reward as it passes by to obtain the larger reward (Bramlett et al., 2012). Intertemporal choice tasks, another example of commitment choice tasks, involve subjects choosing between two differently sized reward options, where selecting the smaller reward results in immediate gratification, while opting for the larger reward necessitates waiting for a designated delay period before receiving the food (Addessi et al., 2013; De Petrillo et al., 2015; Stevens and Mühlhoff, 2012). It is important to note that sustained choice tasks and commitment choice tasks appear to assess distinct facets of delay tolerance. The performance in intertemporal choice and accumulation tasks can differ within the same individuals, highlighting the nuanced nature of the delay of gratification (Addessi et al., 2013; Bramlett et al., 2012). There is debate as to whether commitment choice tasks genuinely assess delay discounting or rather reflect choice preferences (Addessi et al., 2013; Paglieri et al., 2015; Reynolds and Schiffbauer, 2005).

The present study is a continuation of our prior involvement in the ManyPrimates 2 (MP2) data collection within the broader ManyPrimates project – an international collaborative effort dedicated to unravelling the complexities of primate cognition (for details, visit https://manyprimates.github.io/, last access: 11 February 2024; Altschul et al., 2019; ManyPrimates et al., 2021). MP2's primary objective was to conduct a cross-species and cross-site comparison of delay-of-gratification capabilities, using a standardized protocol. Among the tasks employed for this purpose, the exchange task stood out for its simplicity, requiring no specialized equipment or extensive training, making it an ideal choice for comparing performance across diverse groups. Before embarking on the current study, we contributed data from seven long-tailed macaques to the MP2 exchange task. The subjects received a high-value food item that could be exchanged for more food after holding the first food item for a delay period. Each subject was tested in four sessions consisting of 12 trials (exchange opportunities) each. The long-tailed macaques performed surprisingly poorly, despite their familiarity with token exchange. This finding raised concerns that the MP2 protocol might not accurately capture their delay-of-gratification abilities. Specifically, we suspected that the possession of a high-value food item during the delay period might have hindered their performance in the subsequent exchange task. There is extensive literature on the effect of stimulus salience, relative reward value difference, and delay period on delay-of-gratification performance (e.g. Addessi et al., 2014; Auersperg et al., 2013; Beran and Parrish, 2021; Boysen et al., 1996; De Petrillo et al., 2015; Dufour et al., 2007; Evans et al., 2012; Gazes et al., 2018; Genty et al., 2004; Labuschagne et al., 2017; Leonardi et al., 2012; Pelé et al., 2010; Ramseyer et al., 2006; Stevens et al., 2005). We provide an overview of this literature in the Supplement.

During the MP2 data collection, the long-tailed macaques of our study group only ever exchanged anything in one instance. This finding was surprising because they were already well familiar with token exchange procedures as part of other experiments, and they also frequently attempted to initiate exchanges with researchers outside of test sessions (including offering food items from within their enclosure). We were thus sceptical that their performance in the MP2 study was really representative of their ability to delay gratification in the different waiting periods. The aim of this study was to shed light on potential reasons for the poor delay-of-gratification performance of our subjects in the MP2 project. In addition, the results could more generally inform whether reducing inhibitory control demands can scaffold learning in long-tailed macaques.

In the current study, we compared a *high-value* condition (similar to the MP2 exchange option) with a new *mixed-value* condition, in which the food item in possession was of lower quality compared to the high-value condition and the MP2 procedure. In both the high-value and mixed-value condition, the experimenter handed one food item to the monkeys which they could exchange against three high-quality food items after a certain delay period. In the high-value condition, the subjects received one piece of grape (similar to the MP2 test session), and in the mixed-value condition they received a piece of bell pepper, which could then be exchanged for three pieces of grape. We expected that the monkeys would perform better in the mixed-value condition, irrespective of delay period. A consequence of providing the monkeys with a lower-value item was that the *relative* value of the delayed option inevitably increased. To disentangle the effects of stimulus salience and relative reward disparity, a third condition was theoretically required, where both the smaller and larger delayed options would be reduced in salience. However, acknowledging constraints of our limited sample size ( n=6 ), we opted not to include this third condition to ensure a robust experimental design with minimal potential for unwanted carry-over effects. While we will not be able to unequivocally decide if the predicted performance increase was due to reduced salience of the token food item or due to an increase in relative value difference, we will be able to say that these mechanisms potentially play a role in explaining the low performance in the MP2 project. In line with findings of Drapier et al. (2005), we also expected that the probability to exchange decreases with increasing delay period and increases with increasing session number. The difference in performance between high- and mixed-value conditions should decrease with increasing delay period. In the high-value condition, we expected a steeper increase in performance with session number because experience in the mixed-value condition could lead to carry-over effects and influence the performance in high-value sessions. The steepness with which the performance increases with session number will increase more in shorter delay periods compared to longer delay periods.

## 2 Methods

### 2.1 Subjects

We tested six long-tailed macaques in a test room adjacent to their main enclosure. Three subjects were female (mean age 47±30.1 months), and three were male (mean age 36.7±26.7 months). They lived in a social group of 12 individuals with access to an indoor (49 m ${}^{2}$ ) and outdoor (141 m ${}^{2}$ ) enclosure at the German Primate Center in Göttingen, Germany. Our research did not interfere with the monkeys' feeding regime. They received their regular portions of vegetables, fruits, and monkey chow two times per day, and water was available ad libitum. Test subjects entered the test cage voluntarily from their main enclosure and stayed in visual and audible contact with the group. The subjects were tested in morning and afternoon test slots (10:00–12:00 and 14:00–18:00 respectively; time zone: CET until 27 March and CEST after this date). The study was approved by the ethics committee of the Animal Welfare Body of the German Primate Center (permit number E5-21), prior to data collection.

### 2.2 Procedure

In the test phase, each subject received 18 test sessions consisting of 12 trials each. In each test trial, subjects received a small reward (one item) from the experimenter that could be exchanged for a large reward (three items) after a certain delay period. In the high-value condition, subjects exchanged one high-value food item (one small piece of grape) for three pieces of grape, and in the mixed-value condition subjects received one low-value food item (one small piece of bell pepper), which they could exchange for three small pieces of grape. We ensured that pieces of grape and bell pepper were similar in size. The choice of low-value food type was based on a food preference test between different vegetables and grapes. We chose bell pepper because all subjects would eat it when offered alone, but clearly preferred grapes over bell pepper when given the choice. We also ensured that all subjects preferred three pieces of grape over one piece of grape and over one piece of bell pepper and that they understood the exchange procedure (for more details on these preparatory steps, see the Supplement).

Prior to the present project, all subjects participated in the MP2 procedure. The MP2 procedure tested the ability to delay gratification in different delay periods. Table 1 gives an overview of the test phases. The subjects first experienced the high-value quantity preference test, which ensured that all subjects preferred three pieces of grape over one piece of grape. Afterwards, they experienced a familiarization phase where we ensured that all subjects understood the procedure and learned that there was an opportunity to exchange. This step was followed by four MP2 test sessions. Each test session started with the lowest delay period (2 s), and once a subject successfully exchanged their food item in two consecutive trials of the same delay period, the delay was increased (for more details, see https://manyprimates.github.io/mp2/, last access: 11 February 2024, and the Supplement). Following the MP2 data collection, the monkeys experienced four *no-delay* sessions in which they were able to exchange one piece of grape for three pieces of grape without a delay period. Eventually, the subjects experienced the mixed-value quantity preference test and the test phase of the present project. For the current study, a test session consisted of 12 trials of the same delay period and reward value condition. All sessions were tested continuously from January to June 2022.

**Table 1:** Overview of the testing sequence. This table lists the test phases and session types that correspond to the present project as well as the MP2 sessions. The no-delay sessions were not part of the initial MP2 protocol, but we included these sessions to assess whether subjects would exchange in the same procedure without delay.

	Number		
	of sessions		
Phase	per test phase	Delay period	Description
Preparation (MP2)	Two to six	No delay	Quantity preference test High value: ensuring subjects prefer three pieces over one piece of grape
	Two to four	2 s	Familiarization with exchange procedure: ensuring subjects know how to exchange a non-edible token for a small piece of grape
Test (MP2)	Four	2, 5, 10, 20, 40, 80 s	MP2 test sessions: assessing exchange rates with increasing delay periods (one vs three pieces of grape)
Control	Four	No delay	No-delay sessions as a control for MP2 test sessions: assessing exchange rates with no delay (one vs three pieces of grape)
Preparation	Two to six	No delay	Quantity preference test Mixed value: ensuring subjects prefer three pieces of grape over one piece of pepper
Test	Six	2 s	High- and mixed-value test sessions are presented in a pseudo-random order
	Six	4 s	High- and mixed-value test sessions are presented in a pseudo-random order
	Six	8 s	High- and mixed-value test sessions are presented in a pseudo-random order

Each subject experienced 18 test sessions within the present study in total. We presented them with three different delay periods. Delay conditions were presented block-wise: In the first block, the monkeys received six sessions with 2 s delay, in the second block six sessions with 4 s delay, and in the third block six sessions with 8 s delay. In each block, all subjects experienced three sessions of the mixed-value condition and three sessions of the high-value condition. The order of sessions within one block was pseudo-randomized for each subject, whereby the same session type was not presented more than twice in a row. Across all subjects and within each block, both conditions were presented in each session to at least two of the subjects.

**Figure 1:**
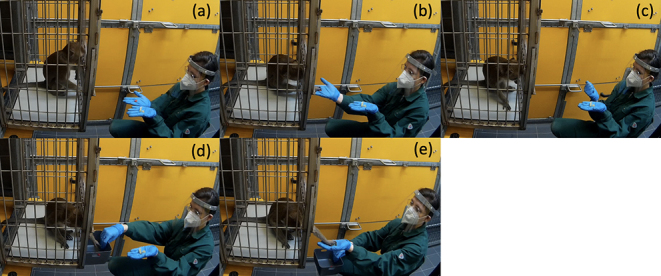
Testing procedure in one trial of the test session. **(a)** Experimenter shows both food options. **(b)** One food item is given to the subject. **(c)** Delay period. **(d)** Exchange of the food item. **(e)** Subject is rewarded with three food items after a successful return.

In each test session, subjects could exchange one piece of food for three pieces of food. The experimenter showed the reward options in open palms out of reach for the subject. The food options were presented in the left and the right hand in a pseudo-randomized manner such that each option was presented equally often on each side but no more than two times consecutively on the same side. Once the subject was attentive, the experimenter moved the hand containing the small reward forward and handed the item to the subject. The empty hand was closed to a fist during the delay, while the large reward was still visible in the other palm. If the subject ate the small reward or touched the piece of food with the mouth during the delay period, the trial ended immediately, and both hands were removed. If the small reward was still intact at the end of the delay period, the experimenter offered an empty basket and allowed the subject to exchange the food. If the subject placed the small reward into the basket within 20 s, the experimenter handed the large reward to the subject. If the subject did not return the small reward within 20 s, the next trial started as soon as the subject had finished eating the current piece of food (see Fig. 1 for the testing procedure in one trial). At the beginning of every test session, we presented two no-delay trials, in which one piece could be exchanged for three pieces without a delay, prior to the test trials. The no-delay trials were presented in the mixed- or high-value condition, depending on the condition of the current session. The no-delay trials should remind the subjects that we offered an exchange possibility in this specific testing setting. This reminder was especially relevant for subjects that did not enter the test cage daily and have more time between the sessions.

### 2.3 Coding and analysis

#### 2.3.1 Coding

As a measure of task performance, we assessed whether the subject successfully returned the complete food item to the basket. We coded cases where the exchange item touched the monkey's mouth as *no exchange*. Cases in which the exchange item was neither eaten nor exchanged were coded as NA. A person who was blind to the hypothesis of the study coded 21 % of the trials. For determining inter-rater reliability, we considered NAs a valid code. Inter-rater reliability was excellent, with Cohen's kappa ( κ)=0.899 (Cohen, 1960).

#### 2.3.2 Analysis

The data were analysed in R (Version 3.6.2, R Core Team, 2020) using a generalized linear mixed model (GLMM) with binomial error structure and logit link function using the lme4 package in R (Baayen et al., 2008; Bates et al., 2015) to estimate the effects of value, delay, and session number on the exchange behaviour (successful exchange yes/no). As we expected the effect of delay and session number to differ between the value conditions and the effect of session number and value to differ between delay periods, we included a three-way interaction between them in the model. Trial number and the age of the subjects were included in the model as control factors, and subject was included as a random intercept effect. We excluded sex as a control factor because we did not expect any influence on the performance. To keep type I error rate at the nominal level of 0.05, we included random slopes (Barr, 2013; Schielzeth and Forstmeier, 2009) of the interaction between the value, delay, and session number, as well as the trial number.

Prior to fitting the model, age, session, delay, and trial were z -transformed to a mean of 0 and a standard deviation of 1, for an easier interpretation of the estimates (Schielzeth, 2010). The variable value was dummy-coded. Due to a “singular fit” message suggesting some of the random effects terms to be unidentifiable, we excluded the estimates of the correlations between random intercept and slopes. Comparing the model lacking the correlations and the model including the correlations revealed a substantial difference (log-likelihoods: full model including correlation parameters: - 265.707; full model lacking correlation parameters: - 284.988). Therefore, we continued with the model including the estimates of the correlations. To test whether the main effects influence the response and to avoid “cryptic multiple testing” (Forstmeier and Schielzeth, 2011), we compared the full model with a null model lacking the main effects using a likelihood ratio test (Dobson and Barnett, 2018). We checked the assumptions of a GLMM by assessing the BLUPs (best linear unbiased predictors). Model stability was estimated by dropping the individuals one at a time from the data and comparing the estimates derived for models to these subsets with those obtained for the full data set. To check collinearity among predictors, we determined variance inflation factors (VIFs; Field, 2005) based on a model lacking the interaction. No collinearity between predictors could be observed (maximum VIF: 1.005). To obtain confidence intervals of model estimates and fitted values, we used a parametric bootstrap (bootMer function of the lme4 package; N=1000 bootstraps). The effect of individual fixed effects was tested by comparing the full model with reduced models lacking them one at a time. A number of R functions were sourced at various stages of the analysis (Mundry, 2023).

We found that subjects neither ate nor exchanged food items in 140 out of 1224 cases (from here on we refer to those NAs as refusals). Since this presents a substantial proportion of the data (11.4 %), we wanted to investigate under which conditions these refusals occur. The long-tailed macaques refused to eat or exchange food items more often in the mixed-value condition compared to the high-value condition. Further, it seems that the session number did not influence the refusals to eat or exchange food items. We tested whether the occurrence of refusals was affected by the value and delay period. As there were only very few refusals to exchange or eat the food item in the high-value condition, a generalized linear mixed model approach was not applicable. Instead, we calculated mean probabilities for refusal for each subject in high- and mixed-value conditions and tested whether the probabilities differ between conditions. Moreover, to assess whether probability of refusal differed between the delay periods, we investigated the refusals in the mixed-value condition and calculated a mean refusal probability per subject and delay period. This post hoc analysis constitutes a data-driven approach that creates a multiple testing problem, which leads to increased type I error rates (Schielzeth and Forstmeier, 2009). Therefore, its interpretation must be taken with caution.

Additionally, we investigated the performance in the two no-delay trials that the subjects received prior to each test session to remind them of the procedure. We built a generalized linear mixed model with binomial error structure and logit link function using the lme4 package in R to analyse whether subjects exchanged more often in the mixed value than in the high-value no-delay trials (Baayen et al., 2008; Bates et al., 2015). The response variable included a matrix with two columns, the number of exchanges in the no-delay trials of a session and the number of no-delay trials in which the subjects did not exchange. The fixed-effect part consisted of the value, which was included as a factor of interest, and the session, which was z -transformed and included as a control factor. The value and session were included as random slopes within the random intercept of the subject, and each individual session was also included as a random intercept.

## 3 Results

### 3.1 Delay-of-gratification performance

**Figure 2:**
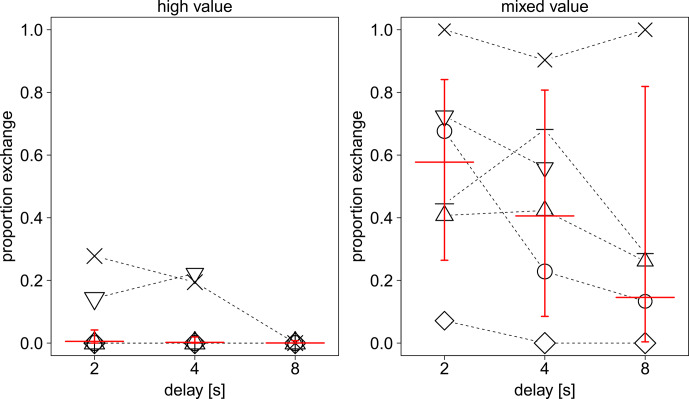
Performance in high- and mixed-value conditions for all delay periods. Each data point represents the proportion of successful exchanges in three sessions. The area of the symbols (or the area it circumscribes) is proportionate to the sum of trials within three sessions (range = 27–36). Each marker type connected with dotted lines represents an individual subject. Red lines show the fitted model (full model) and its confidence limits. All other terms of the model are centred to a mean of 0.

**Figure 3:**
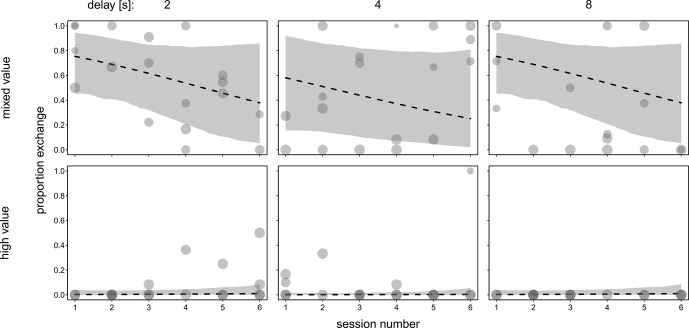
Performance in mixed- and high-value conditions with 2, 4, and 8 s delay. Each data point represents the proportion of successful exchanges of one session of one individual. Due to the pseudo-randomized order of the conditions for each subject, each session number contains data points of two to four subjects (both conditions were presented in each session to at least two of the subjects). The shades of grey show overlapping data points, and the area of the dots is proportionate to the number of trials per session (range = 2–12). Dotted lines indicate the fitted model (additive model) and the grey area its confidence limits. All other terms of the model are centred to a mean of 0.

Overall, the long-tailed macaques' performance increased in the present project compared to the MP2 session (see MP2 performance in Fig. S1 in the Supplement), and the tested subjects were indeed able to delay gratification. Descriptively, this performance increase was mainly driven by the new mixed-value condition (46.43 % exchanges), whereas performance in the high-value condition was much more similar to the monkeys' exchange rate in MP2 (MP2: 0.30 %, high-value condition: 7.41 %). To investigate the effect of reward value on the task performance, we conducted 1224 test trials. We excluded 140 cases in which the subjects neither exchanged nor ate the food item. The remaining 1084 test trials were included in the model. The full model including the three-way interaction between value, session, and delay explained significantly more variance in the data than the null model (likelihood ratio test: $\chi^{2}=17.751$ , d*f* = 7, p=0.013 ). The three-way interaction did not contribute significantly to explaining variance in the full model (see Table S3). Therefore, we ran a reduced model comprising all two-way interactions but lacking the three-way interaction (see Table S4). None of the two-way interactions were significant, so they were dropped from the model, resulting in an additive model comprising only the main effects (see Table 2). We found significant effects of value and delay period: 36.2 % of the variance was explained by the fixed factors and 50.7 % by the fixed and random effects of the full model (MuMIn package, Barton, 2020). We found that performance increased in the mixed-value condition compared to the high-value condition. Moreover, the results indicate that performance decreased with increasing delay period. Figure 2 shows the effects of condition and delay with respect to individual differences. Figure 3 shows the effect of session number in both conditions. Individual differences in performance are shown in Fig. S4 in the Supplement.

**Table 2:** Results of the additive model, which investigated the effects of value, delay, and session on performance in the delay-of-gratification exchange task. (Estimates, together with standard error (SE), confidence limit (Cl), significance tests ( P ), and the range of estimates obtained when excluding cases one at a time (min, max).)

Term	Estimate	SE	Cl (lower)	Cl (upper)	P	Min	Max
Intercept	- 6.612	1.983	10.465	- 4.585	${}^{a}$	- 7.824	- 5.580
Value (mixed value)	6.078	1.656	4.722	8.746	0.000	5.581	7.163
Delay ${}^{b}$	- 0.803	0.374	- 1.875	- 0.130	0.032	- 1.054	- 0.542
Session ${}^{c}$	- 0.423	0.378	- 1.207	0.308	0.263	- 0.716	0.007
Trial ${}^{d}$	- 0.202	0.184	- 0.578	0.186	0.273	- 0.313	- 0.129
Age ${}^{e}$	1.043	0.654	- 0.203	2.616	0.111	0.598	4.320

${}^{a}$ Not indicated due to limited interpretability. ${}^{b}$ z -transformed to a mean of 0 and a standard deviation of 1; original mean and standard deviation were M=4.481 and SD = 2.443. ${}^{c}$ z -transformed to a mean of 0 and a standard deviation of 1; original mean and standard deviation were M=3.511 and SD = 1.691. ${}^{d}$ z -transformed to a mean of 0 and a standard deviation of 1; original mean and standard deviation were M=6.608 and SD = 3.444. ${}^{e}$ z -transformed to a mean of 0 and a standard deviation of 1; original mean and standard deviation were M=40.012 and SD = 23.681.

### 3.2 Refusal behaviour (post hoc analysis)

**Figure 4:**
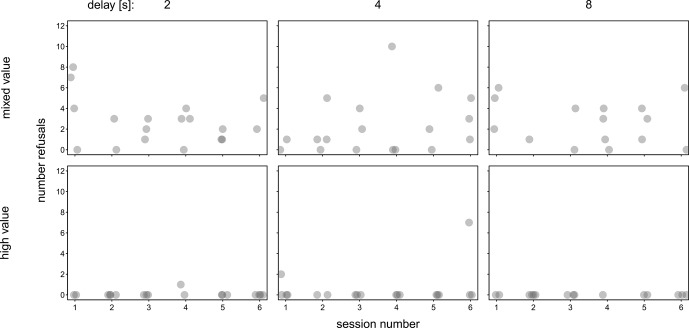
Number of refusals per session. Each data point comprises the number of NAs of one session. The shades of grey show overlapping data points. Due to the pseudo-randomized order of the conditions for each subject, each session number contains data points of two, three, or four subjects (both conditions were presented in each session to at least two of the subjects).

The post hoc analysis of refusals in the test sessions (trials in which subjects neither ate nor exchanged food items) was conducted based on the investigation of Fig. 4, which depicts the number of refusals in each session. An exact Wilcoxon signed-rank test revealed that the monkeys refused to exchange food items significantly more often in the mixed-value condition compared to the high-value condition ( T=21 , p=0.031 ), with more refusals occurring in the mixed-value condition. Further, a Friedman rank sum test revealed no significant difference of refusal rates between the delay periods ( $\chi^{2}=2.632$ , d*f* = 2, p=.268 ).

### 3.3 No-delay trials

**Figure 5:**
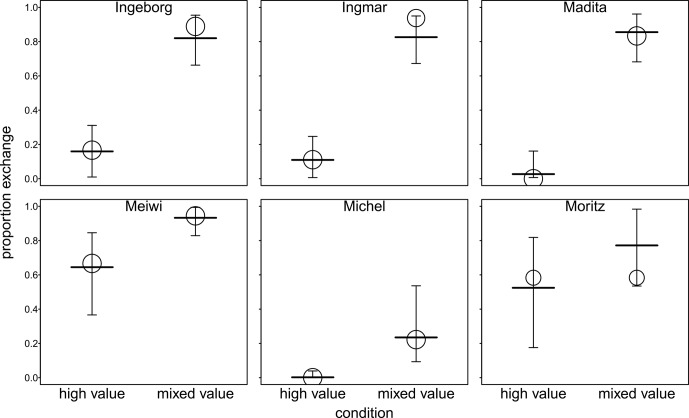
Performance in no-delay trials prior to each test session in the respective condition per subject. Before each test session, subjects received two no-delay trials in the respective condition. The points depict the proportion of exchanges of each subject, and their area is proportional to the number of sessions they depict (range = 6–9). Horizontal lines present the individual fitted values for each subject, and the vertical lines show the respective confidence intervals.

Subjects' performance in the no-delay trials is depicted in Fig. 5. The probability to exchange in the no-delay trials prior to each test session was analysed using a generalized linear mixed model (model results can be found in the Supplement, Table S5). Dropping the fixed effects from the full model one at a time using the “drop one” function revealed that the long-tailed macaques exchanged more often in the no-delay trials of a mixed-value condition session than of a high-value condition session ( p=0.007 ).

## 4 Discussion

We found that delay-of-gratification performance of six long-tailed macaques in an exchange task was enhanced when the food item in possession was of low value compared to high value. In our study, we assessed spontaneous delay-of-gratification ability rather than maximum performance after extensive pre-training. The monkeys regularly endured delays of up to 4 s but only seldom waited 8 s to exchange their food item for the larger delayed option. This finding stands in contrast to the same individuals' performance in the ManyPrimates (MP2) data collection, where their delay-of-gratification performance was almost non-existent. Crucially, in the MP2 protocol, the item in possession was always of high-value quality. These results may indicate that having a high-value food item in possession induced a mental distraction for these individuals, diverting their attention from the otherwise familiar exchange task protocol and hampering their ability to sustain any delay period. Alternatively, increased delay-of-gratification performance in the mixed-value condition may be the consequence of an increased relative value difference between the item in possession and the exchange option. As alluded to earlier in the Introduction, the current study cannot unequivocally distinguish between these possibilities.

Importantly, we want to emphasize that our intention is not to insinuate any inherent flaws in the MP2 protocol, nor do we suggest that it had a similar impact on other study populations. In fact, prior research has indicated that long-tailed macaques may be prone to distraction and exhibit poorer inhibitory control abilities in comparison to other primates (Amici et al., 2008). However, with this study we could show that they can exhibit patience when their needs (reduced cognitive distraction) are adequately met. In these findings, the challenge of distinguishing between competence and performance becomes apparent: successful performance during assessment of a certain cognitive domain may differ greatly depending on situational variables such as stimulus material, task, instructions, context, or the experimenter (see, for example, Flavell and Wohlwill, 1969; Wood and Power, 1987); hence the limits of any generalization claim have to be explored carefully (e.g. Rogoff, 1981). Although making claims about the presence or absence of an individual's or species' cognitive abilities has gained popularity in scientific publishing, we should not lose sight of treating an ability as something with a range of possible values which may vary with situational factors. As became apparent from our study as well as a large body of literature using different tasks, rewards, and procedures, delay-of-gratification performance in primates and other animals varies greatly based on the rewards involved and the available alternatives (for a brief overview, see Supplement). Despite such procedure-dependent variation, it will be very interesting to see and compare performance patterns of a large number of primates who have been tested with identical procedures, once MP2 data collection is resumed and the results are analysed.

Contrary to our initial expectations, we observed neither an increase in delay-of-gratification performance with increasing experience nor a carry-over effect from mixed-value to high-value conditions. All subjects exhibited the ability to exchange food items during their first session in the mixed-value condition, even in the absence of prior training sessions involving edible items. This clear demonstration of comprehension of the test procedure indicates that our subjects had a firm grasp of the task's mechanics. Had extensive training been a prerequisite for assessing delay-of-gratification abilities, we would have observed a generally low performance initially, with an incremental improvement in exchange rates as sessions progressed, particularly within the mixed-value condition. Thus, we can reasonably conclude that our experiment effectively measured spontaneous delay-of-gratification performance with those specific rewards. The prior low performance observed in high-value sessions (MP2) appears to stem from heightened inhibitory control demands rather than a lack of understanding. It is worth noting that certain studies have employed substantially lengthier training procedures, during which subjects gradually learned to endure delays more effectively (Addessi et al., 2011; Pelé et al., 2010). Such findings led us to initially hypothesize that performance might improve with increasing experience in the current study. However, our findings indicate that experience from previous sessions did not translate into enhanced performance in subsequent sessions within the same delay block.

The long-tailed macaques exhibited a refusal to exchange their food item (neither consuming nor exchanging it) in approximately 11 % of the cases, mostly in the mixed-value condition. This substantial rate of refusals could potentially suggest that the low-value food (bell pepper) was not enticing enough for the subjects, leading them to opt not to exchange it and, instead, perhaps discard it elsewhere within the test cage. It is also possible that the high rate of refusals might be related to the relatively conservative coding criterion agreed upon by the MP2 project group. Specifically, a refusal was coded when the subject dropped the food item outside the test cage without placing it into the offered basket or when they touched the food item with their lips or mouth. Previous research has shown that capuchin monkeys' performance in exchange tasks significantly improved when subjects were allowed to nibble on the food item before making an exchange (Drapier et al., 2005; Ramseyer et al., 2006). Even though it is not possible to assess this possibility with the data from the present study, it would be interesting to test whether the observed performance difference between the mixed- and high-value conditions persists when applying a less conservative success criterion.

The finding that performance increased with reduced value of the food item in possession may have useful implications for training contexts. In inhibitory control training settings, the use of lower-value food items can function as an intermediate training step and help to speed up training success. In Peleí et al. (2010), long-tailed macaques were trained to delay gratification in an exchange task. The monkeys first exchanged low-value food items for high-value food items as a training step to exchange different food quantities later in the test sessions. The present study supports this approach as we show that using a reduced reward value in an inhibitory control task can facilitate performance in the task. When aiming for smaller training steps, this can be a valuable approach to train animals.

In summary, we compared delay-of-gratification performance of long-tailed macaques in two conditions that differed in terms of the quality value of a food item that the monkeys could exchange for a higher-value reward. We found that reducing the value of the item in possession led to an increase in delay-of-gratification performance. In line with a considerable body of existing literature documenting variation of cognitive performance as a function of procedural and situational parameters, the current findings illustrate the importance of interpreting the results of animal cognition experiments in the light of the context in which data were collected rather than as generic abilities at the individual or species level.

## Supplement

10.5194/pb-11-19-2024-supplement The supplement related to this article is available online at: https://doi.org/10.5194/pb-11-19-2024-supplement.

## Data Availability

Data and code are available on OSF (10.17605/OSF.IO/Z49EF, Stolla and Keupp, 2024).

## Video supplement

Example videos of the test sessions and preparatory steps are available on OSF (https://doi.org/10.17605/OSF.IO/Z49EF, Stolla and Keupp, 2024).
