# Supplementary material for: The effect of reward value on the performance of long-tailed macaques (Macaca fascicularis) in a delay-of-gratification exchange task
Source: Primate Biol. 2024 Jul 26;11(2):19–29. doi: 10.5194/pb-11-19-2024 (PMC12824853; doi:10.5194/pb-11-19-2024)
Supplement: The supplement related to this article is available online at: https://doi.org/10.5194/pb-11-19-2024-supplement. [file pb-11-19-2024-supplement.pdf]

Supplement of Primate Biol., 11, 19–29, 2024
https://doi.org/10.5194/pb-11-19-2024-supplement
© Author(s) 2024. CC BY 4.0 License.

Primate Biology
Open Access

Supplement of

The effect of reward value on the performance of long-tailed macaques (*Macaca fascicularis*) in a delay-of-gratification exchange task

Judit J. Stolla and Stefanie Keupp

Correspondence to: Judit J. Stolla (jstolla@dpz.eu)

The copyright of individual parts of the supplement might differ from the article licence.

Supplementary Information

Literature review

The motivation to explore delay of gratification abilities of our group of long-tailed macaques was sparked by our surprise of their poor performance in a food exchange task, which we conducted in the context of the ManyPrimates project (MP2). Prior to the MP2 data collection, the monkeys were already well familiar with exchanging items with experimenters, including holding them in their hands for several seconds until the experimenter fetched an exchange reward from out of their pockets or a nearby food tray. Hence, we suspected that something in the procedure of MP2 had interfered with their ability to wait for a better reward. Among the possibilities that came to mind were the salience of food exchange options and the relative difference between available options. There is indeed an extensive body of literature on the effects of stimulus salience, relative reward value difference, and delay period on delay of gratification performance, which we briefly review here, as we think it might be of interest for readers who are not yet familiar with the topic.

Stimulus salience

Stimulus salience appears to influence performance in various delay of gratification tasks, but the methods used to reduce salience (e.g., employing less salient food items or non-edible tokens) play a pivotal role. In broad terms, salience can be described as the "property of a stimulus, or of previous experiences associated with a stimulus, that causes an organism to focus its attention toward this stimulus" (Rumbaugh et al., 2007). The operationalization of salience has taken various forms, and these differing methods have yielded varying effects on delay of gratification performance across studies.

In many investigations, subjects faced choices involving high-value food items, all of which possessed high salience due to their rewarding quality (Addessi et al., 2011; Amici et al., 2008; Beran et al., 2016; De Petrillo et al., 2015; Pelé et al., 2010; Rosati et al., 2006; Stevens & Mühlhoff, 2012). In these instances, the tested species demonstrated delay of gratification abilities with high-value food items. However, other studies have revealed that stimulus salience can impact inhibitory control performance under different circumstances. Some of these studies reference salience when examining distinctions between edible and inedible conditions (Addessi et al., 2014; Evans et al., 2012; Judge & Essler, 2013). Replacement of highly salient items can be divided into symbolic and iconic (non-symbolic) replacement. While iconic substitutes have similar properties as the referent, symbols are arbitrary chosen items that do not have any iconic relation to their referent (Addessi et al., 2007). For instance, in an intertemporal choice task, capuchin monkeys exhibited a stronger preference for the

larger, delayed option when choosing between food options compared to tokens (Addessi et al., 2014). The rationale behind this choice was that replacing food with symbolic tokens removed the appetitive characteristics of the stimuli, making the results more interpretable in an intertemporal choice task. Subjects might genuinely display delay aversion only in the token condition, as the choice in the food condition was otherwise dominated by a prepotent response towards the larger option (Addessi et al., 2014). While this explanation pertains specifically to commitment choice tasks, a similar pattern emerged with iconic stimuli in an accumulation task, where capuchins accumulated more food rewards than tokens (Evans et al., 2012). In contrast, chimpanzees did not exhibit any performance differences between food and token conditions. The authors argued that token stimuli did not simplify the complexity of the accumulation task when they represented the quantity dimension (Evans et al., 2012). In an exchange task, subjects were given the choice to exchange a token for either an immediate, medium-value food reward or for a token that could be exchanged for a high-value food reward later. Both capuchin monkeys and chimpanzees consistently opted for the high-value token over the immediate food reward (Beran & Evans, 2012; Judge & Essler, 2013). Consequently, the effect of token usage on delay of gratification tasks appears ambiguous. Symbolic token replacement removes quantitative features that might facilitate a prepotent response (Evans et al., 2012) and necessitates heightened cognitive resources (Beran & Parrish, 2021). However, iconic replacement could decrease salience compared to food items without increasing cognitive load, as the quantity of items remains constant compared to the food condition. Notably, a study involving 4-year-old children found that symbolic, but not iconic, replacement of rewards impaired children's performance in a delay of gratification task (Labuschagne et al., 2017). Additionally, a grey parrot exhibited longer waiting times in an exchange task when the two options differed in quality than when they differed in quantity. Subsequently, when food items were replaced with iconic tokens, the parrot performed similarly to the quality task and better than in the quantity exchange task (Koepke et al., 2015; Pepperberg & Rosenberger, 2022).

The impact of tokens on performance was also investigated in reverse-reward contingency tasks, which assess inhibitory control. In these tasks, subjects must choose the smaller of two quantities to receive the larger one (Boysen et al., 1996; Boysen & Berntson, 1995; Genty et al., 2004; Genty & Roeder, 2007; Kralik, 2005). Capuchins' performance in a reverse-reward contingency task improved when choosing between symbolic tokens but not when choosing between iconic tokens compared to food items (Addessi & Rossi, 2011). Chimpanzees could not consistently select the smaller of two quantities until food quantities were replaced by Arabic numerals (Boysen & Berntson, 1995), with similar results found in children (Carlson et al., 2005). Furthermore, effects of iconic replacement in primates have been observed in number discrimination studies. Schmitt and Fischer (2011) reported improved performance in long-tailed macaques and olive baboons when tested with inedible items

compared to edible items. When subjects discriminated between food quantities but were rewarded with different food items, their performance was equally high as in the inedible condition. This pattern suggests that not only salience but also the internal representation plays a role in performance (Schmitt & Fischer, 2011). However, Gazes and colleagues (2018) could not replicate this finding in capuchin monkeys, who chose the larger quantity more frequently in the edible condition than in the inedible and edible replaced condition. In a second experiment of this study, the authors increased the subjective value of the food options by replacing the food items with a more preferred food type while keeping the same quantity items in the respective options. This manipulation led to enhanced performance in comparison to using a less preferred food type, which the authors attributed to potentially heightened motivation (Gazes et al., 2018). Moreover, in an exchange task, dogs were increasingly influenced by the relative size of the food reward as they approached their maximum waiting time (Leonardi et al., 2012). The dogs were more inclined to exchange one low-value food item for a higher-value food reward rather than settling for low-value returns. In summary, reducing stimulus salience through tokens can alter primates' performance, but results vary across studies. Symbolic tokens may increase task difficulty, while the impact of iconic replacement remains less clear.

Relative value difference

By diminishing the value of the food item held in possession, we effectively alter the relative value discrepancy between the smaller and larger delayed rewards. Apart from specific factors such as an individual's current satiety or food aversions, the value of a food reward is generally determined by both its quantity and quality (Rachlin, 1971), and both of these dimensions influence the relative value among various reward options. It has been suggested by De Petrillo and colleagues that non-human primates may exhibit a heightened sensitivity to quality over quantity (De Petrillo et al., 2015). For instance, in their successful training of long-tailed macaques to endure delay periods within an exchange task, Pelé and colleagues (2010) introduced a quality condition as a preparatory step before transitioning to the quantity exchange task. Similarly, capuchin monkeys showed increased performance in an exchange task, when the relative value difference increased and when options were differentiated by quality rather than quantity (Drapier et al., 2005; Ramseyer et al., 2006). This distinction between quantity and quality conditions has been evident in various species. New Caledonian crows and children, for instance, exhibited improved performance in a rotating tray task when the reward differed in quality as opposed to quantity (Miller et al., 2020). Interestingly, the crows' performance was further boosted when they encountered the quality condition first. Similarly, the performance of Goffin cockatoos, ravens, and carrion crows improved in the quality condition of a delay of gratification exchange task compared to the quantity condition (Auersperg et al., 2013; Bugnyar et al., 2012; Hillemann et al., 2014).

Beyond the distinction between quantity and quality conditions, the magnitude of the larger delayed reward also plays a significant role in performance. The magnitude effect posits that the rate of discounting varies systematically depending on the size of the delayed reward (Green et al., 1997, 1999; Kirby, 1997; Kirby & Maraković, 1996). Specifically, smaller options are subject to steeper discounting rates than larger ones. While some researchers have suggested that this effect is consistent primarily in humans (Green et al., 1999; Green & Myerson, 2004), it has also been observed in pigeons (Grace et al., 2012). In this intertemporal choice task, the pigeons chose between a small reward which was accessible after short time and a large reward which was accessible after more seconds. When adjusting the delay periods, the researchers found that the temporal discounting rate was decreased for the large reward compared to the small reward (Grace et al., 2012). However, other studies could not consistently confirm the magnitude effect in pigeons and rats (Green et al., 2004; Richards et al., 1997). In scenarios where marmosets and cotton-top tamarins had to decide between traveling to a larger but more distant option or opting for a smaller, nearby option, the ratio of the quantities, rather than the overall magnitude of the rewards, proved decisive (Stevens et al., 2005). In summary, the magnitude, quality, and quantity differences of rewards all hold relevance for performance in delay of gratification tasks. In the current study, by substituting the high-value food item in possession with a low-value (less preferred) food item in the *mixed value* condition, we not only altered its salience but also its quality.

Effects of delay periods and training

The ability to delay gratification also depends on the delay period (e.g., Pelé et al., 2010). Exchange rates of long-tailed macaques and capuchin monkeys were higher when delay periods were shorter (Pelé et al., 2010; Ramseyer et al., 2006). Importantly, subjects were tested in stages with increasing time-lags and the testing ended as soon as the individuals' performance dropped to zero exchanges (Pelé et al., 2010). Similarly, chimpanzees' return rates decreased with increasing time lags in an exchange task. When receiving a 40 times larger reward after the exchange, the performance differed less between time lags of up to 4 minutes than when the delayed reward was 2-, 4-, or 8 -fold the size of the initial food item (Dufour et al., 2007). These findings match the pattern of temporal discounting, where the subjective value assigned to an item decreases with increasing delay period. Primates show temporal discounting effects and species differ in the extent with which they show temporal discounting (Rosati et al., 2006). Therefore, we were interested to learn to what extent the ability to inhibit food consumption during the exchange task decreases with increasing delay period. Additionally, we expected that the long-tailed macaques' performance would increase with increasing experience, as this was a training effect also observed by Pelé and colleagues (2010). In the training phase of Pelé and colleagues long-tailed macaques were trained to exchange edible items without

delay (Pelé et al., 2010). Their performance increased until they reached criterion after 3 to 13 sessions (criterion: succeed in 80 % of the trials during two consecutive sessions (12 trials per session)).

Additional Methods and Results

Table S1 Subject data including the number of test sessions received. Each subject should have received six sessions in each delay block, meaning 18 test sessions in total. Moritz refused to enter the test cage for a long time and therefore, the testing could not be continued after the second block (12 sessions). He never experienced test sessions with an 8-second delay period.

Subject name	Sex	Age (months)	Number test sessions received
Meiwi	female	73	18
Madita	female	54	18
Ingeborg	female	14	18
Moritz	male	65	12
Michel	male	33	18
Ingmar	male	12	18

Food type preference test

In order to find a kind of food item that we could use for the *mixed value* condition, we tested the preferences of seven subjects (Michel, Ingeborg, Ingmar, Lenny, Meiwi, Isabella, Moritz (Lenny and Isabella did not participate in the study; Madita did not participate in the food type preference test but participated in the study)). Five subjects received between 72 and 96 trials, but since Isabella and Moritz entered the test cage so infrequently, we decided to continue with the quantity preference test (they participated in 32 and 48 trials respectively). We tested 524 trials in total across all seven individuals. In each trial, one similar-sized food item was presented in each hand and we assessed which item they chose. At the start of a trial, both palms were moved forward to the subject. The subjects received the food in the palm they first indicated. The other palm was removed as soon as subjects indicated their choice. During each session consisting of 12 trials, the presentation side for the food items was pseudo-randomised such that each food item was presented on each side for the same number of trials. We presented celery vs. bell pepper, celery vs. carrot, carrot vs. bell pepper as well as celery, bell pepper, and carrot each vs. grape. We chose bell pepper, celery, and carrot as these foods are part of the monkeys' daily feeding schedule. We found a preference for bell pepper over celery (bell pepper was chosen in 65.9 % of the 88 trials in total) and carrot (bell pepper was chosen in 53.8 % of the 80 trials in total) and a clear preference for one piece of grape over one piece of bell pepper (grape was chosen in 96.7 % of the 90 trials in total). Therefore, we chose bell pepper as the low value food item for the *mixed value* condition.

Food quantity preference test

The preference test for food quantities aimed to ensure that subjects preferred the delayed option over the exchange item. During this quantity preference test, each subject received a maximum of six sessions with 12 trials each. To reach the success criterion, the subjects had to choose the 3 pieces of

food over 1 piece in 10/12 trials in two consecutive sessions. At the start of a trial, the experimenter presented one piece of food in one palm and three pieces of food in the other palm. Both palms were moved forward to the subject. The subjects received the food in the palm they first indicate. As soon as subjects had indicated their choice, the other palm was removed. During each session, the presentation side of the food was randomized and counterbalanced. Subjects experienced food size preference tests for both conditions: First, they experienced the quantity preference test in the *high value* condition (1 versus 3 pieces of grape) and then they experienced the quantity preference test in the *mixed value* (1 piece of pepper versus 3 pieces of grape). In the *high value* quantity preference test, four subjects reached the criterion straight away, one subject needed three sessions and another one four sessions. In the *mixed value* quantity preference test, all subjects reached the criterion straight away.

Familiarization with exchange procedure

After reaching the criterion in the quantity preference test, each subject exchanged non-food items (tokens) with the experimenter. Here, subjects were tested until they returned the token in three consecutive trials in two consecutive sessions. The token, in this case a piece of coconut shell, was presented in one palm and the small reward (one small piece of grape) was presented in the other palm, both out of reach for the subject. The palm with the token was moved forward and the token was given to the subject. In case the subject did not take the token, the experimenter pushed it into the enclosure. The empty hand was closed and pulled back in line with the other hand containing the piece of food. After 2 seconds, an empty basket was offered to the subject in which the token could be returned. If the subjects returned the token within 30 seconds, they received the piece of food. If the subjects did not return the token, the trial ended. Independent of whether the subjects returned the token in the previous trial, they received a new token at the beginning of the next trial. We presented sessions of twelve trials each. All subjects reached the criterion straight away (n = 7).

Table S2 Differences in the test procedure between the ManyPrimates project and the present project. The protocol was adapted in order to address the research questions of the present project. A consistent number of trials was required to assess a training effect.

	**ManyPrimates Project**	**Present Project**
Conditions tested	High value condition	High and mixed value condition
Presented delay period	Dependent on performance in prior trial (when succeeded in 2 consecutive trials, continue with the next delay period) 2,5,10,20,40,80 s	Fixed delay period in each session 2,4,8 s
Time frame to return food item after delay period ended	30 s	20 s

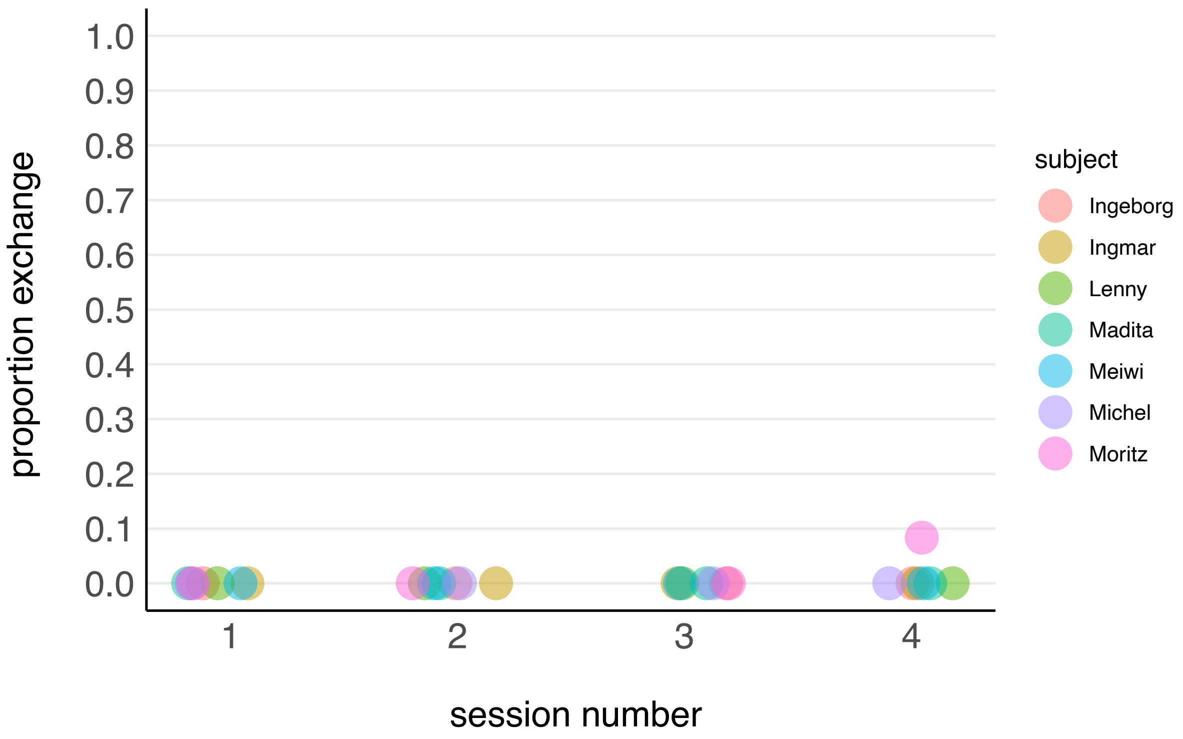

Figure S1 Performance of all subjects in the four ManyPrimates test sessions prior to the data collection for the present project. Sessions consisted of 12 trials with high value food items. Since the subjects did never meet the criterion to continue with longer delay periods, all trials were tested with 2 seconds delay. Details on the protocol can be found here https://manyprimates.github.io. An additional subject (Lenny) was tested in the main enclosure (not in the test cage) in the ManyPrimates sessions because he did not enter the test cage. To exclude methodological variation, Lenny did not participate in the present project.

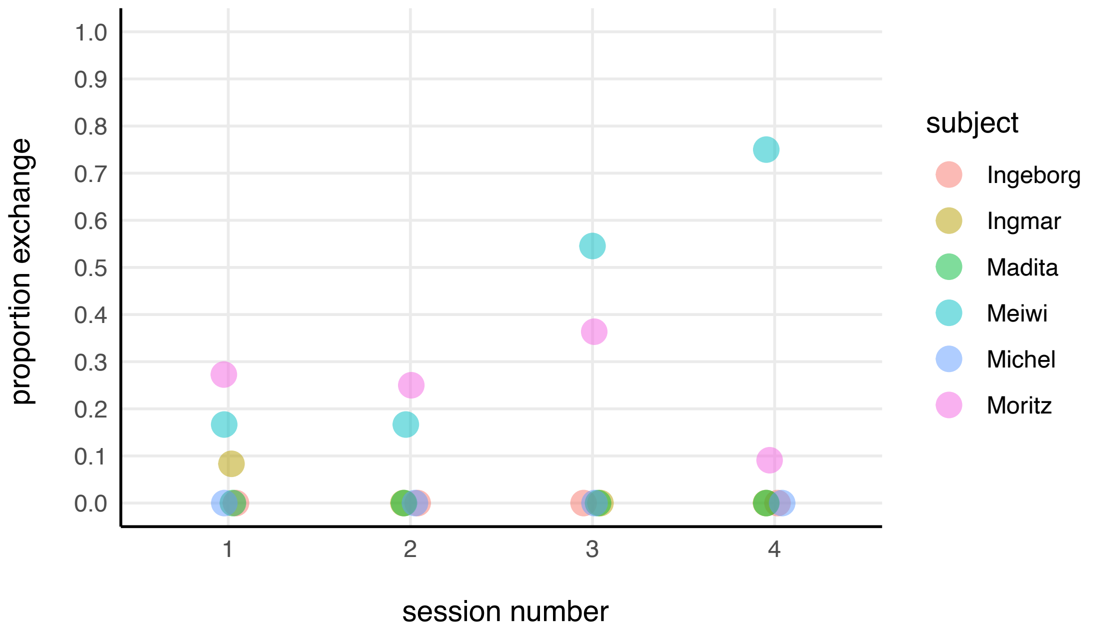

Figure S2 Performance of all subjects in the four no-delay sessions prior to the data collection for the present project. Sessions consisted of 12 trials in the high value condition.

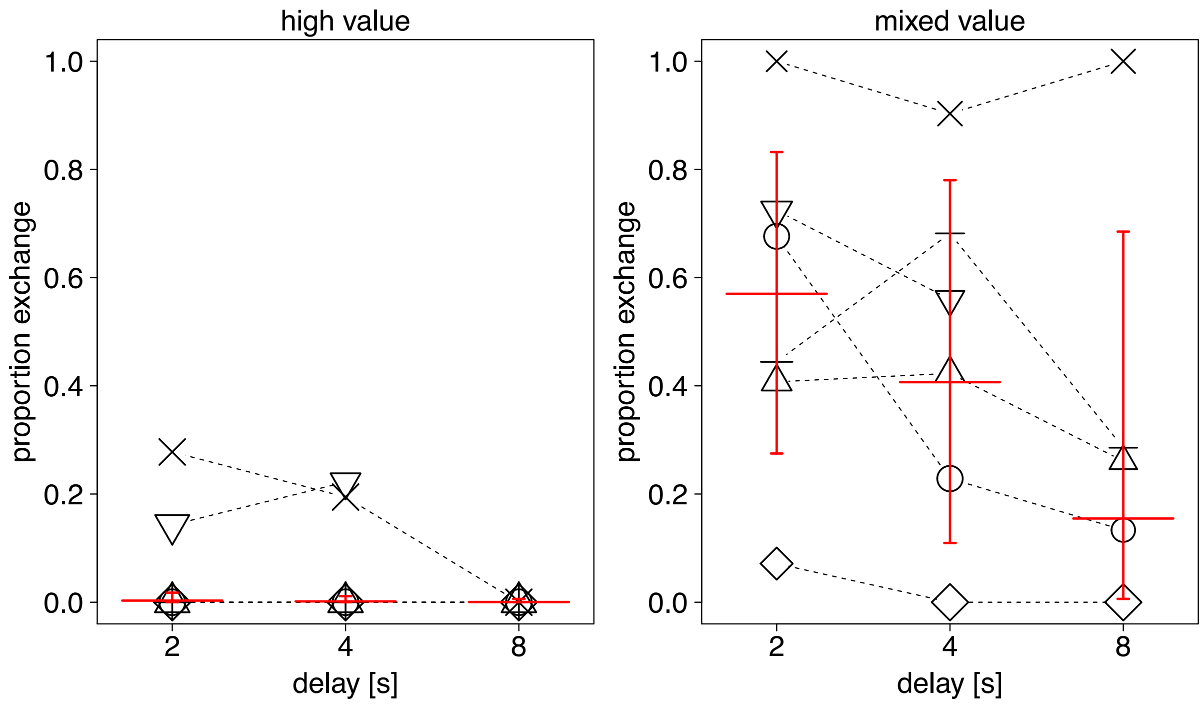

Figure S3 Performance in high and mixed value condition for all delay periods. Each data point represents the proportion of successful exchanges in three sessions. The area of the symbols (or the area it circumscribes) is proportionate to the sum of trials within three sessions (range = 27–36). Each marker type connected with dotted lines represents an individual subject. Red lines show the fitted model (additive model) and its confidence limits. All other terms of the model are centered to a mean of zero.

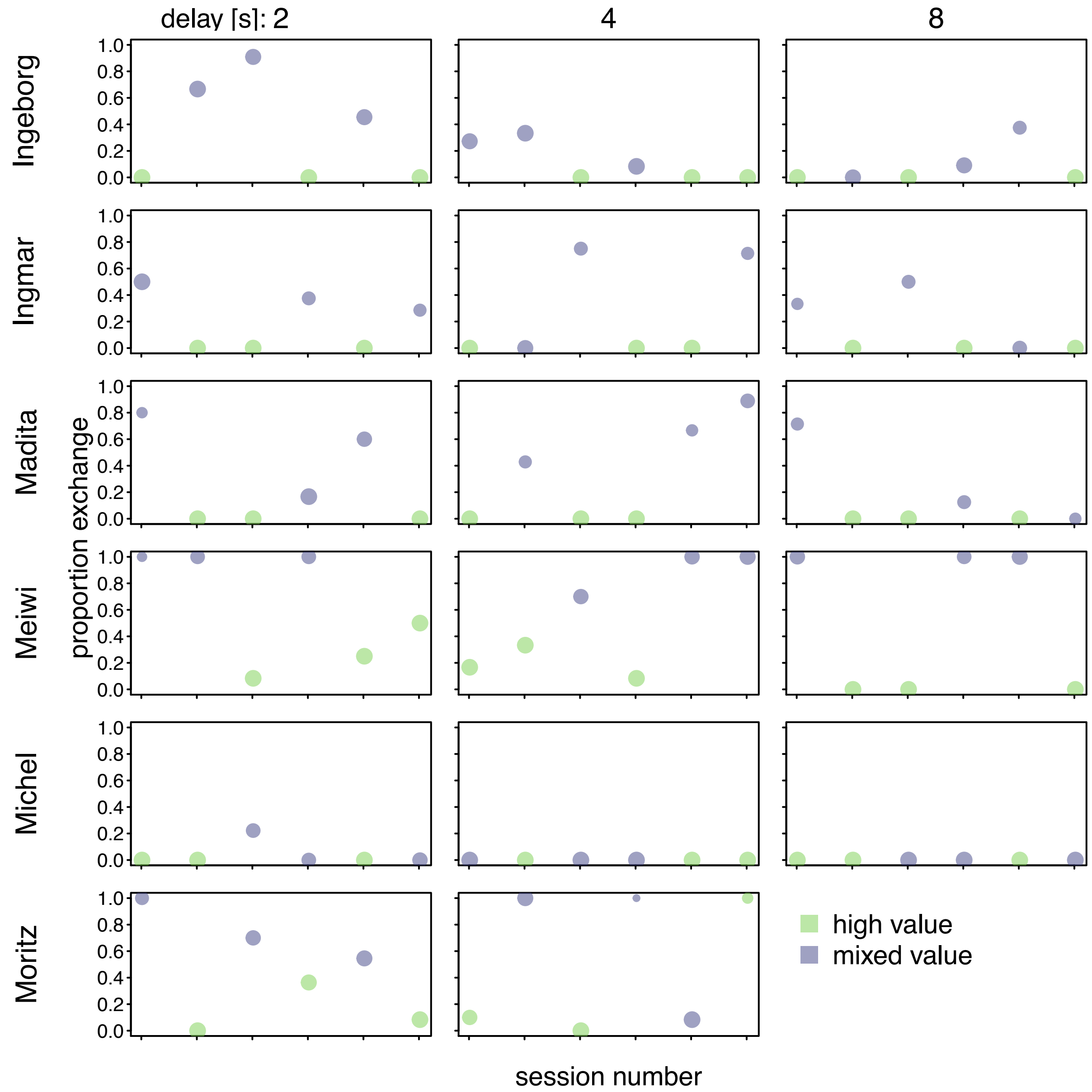

Figure S4 Performance in mixed and high value condition with 2-, 4-, and 8-seconds delay per subject. Each data point comprises the proportion of successful exchanges of one session. The area of the dots is proportionate to trials per session, which ranged from 2 to 12 (due to the high number of NAs in some sessions).

Table S3 Results of model 1 (full model), which investigated the effects of value, delay, session, and all interactions on performance in the delay of gratification exchange task. (Estimates, together with standard errors (SE), confidence limits (Cl), significance tests (P), and the range of estimates obtained when excluding cases one at a time (min, max))

Term	Estimate	SE	Cl (lower)	Cl (upper)	P	min	max
Intercept	-6.296	2.048	-10.344	-4.074	(1)	-8.682	-5.277
Value (mixed value)	5.750	1.865	4.235	8.954	(1)	4.753	8.041
Delay (2)	-1.079	1.583	-3.192	0.711	(1)	-1.673	-0.910
Session (3)	0.323	1.489	-1.260	2.375	(1)	-0.769	0.783
Trial (4)	- 0.195	0.221	-0.646	0.238	0.378	-0.335	-0.008
Age (5)	1.177	0.783	0.038	2.669	0.133	0.605	4.042
Value (mixed value): Delay (2)	0.236	1.663	-1.997	2.365	(1)	-0.225	0.720
Value (mixed value): Session (3)	-0.785	1.747	-3.536	1.373	(1)	-1.365	0.851
Delay (2)*: Session* (3)	-0.117	1.564	-1.690	1.973	(1)	-1.305	0.340
Value (mixed value): Delay (2)*: Session* (3)	0.198	1.757	-2.412	2.170	0.910	-0.370	1.764

(1) not indicated due to limited interpretability

(2) z-transformed to a mean of zero and a standard deviation of one; original mean and standard deviation were M = 4.481 and SD = 2.443

(3) z-transformed to a mean of zero and a standard deviation of one; original mean and standard deviation were M = 3.511 and SD = 1.691

(4) z-transformed to a mean of zero and a standard deviation of one; original mean and standard deviation were M = 6.608 and SD = 3.444

(5) z-transformed to a mean of zero and a standard deviation of one; original mean and standard deviation were M = 40.012 and SD = 23.681

Table S4 Results of model 2 (reduced model) which investigates the effects of value, delay, session, and 2-way interactions on performance in the delay of gratification exchange task. (Estimates, together with standard errors (SE), confidence limits (Cl), significance tests (P), and the range of estimates obtained when excluding cases one at a time (min, max)).

Term	Estimate	SE	Cl (lower)	Cl (upper)	P	min	max
Intercept	-6.270	2.033	-10.425	-4.350	(1)	-8.319	-5.374
Value (mixed value)	5.722	1.830	4.283	9.057	(1)	4.523	7.632
Delay (2)	-1.070	1.542	-3.216	1.266	(1)	-1.405	-0.902
Session (3)	0.444	1.030	-0.626	2.549	(1)	0.047	0.683
Trial (4)	-0.186	0.208	-0.573	0.217	0.371	-0.330	-0.032
Age (5)	1.165	0.764	0.065	2.625	0.127	0.604	4.020
Value (mixed value): Delay (2)	0.241	1.607	-2.627	2.402	0.881	-0.255	0.661
Value (mixed value): Session (3)	-0.937	1.101	-3.368	0.698	0.395	-1.611	-0.417
Delay (2)*: Session* (3)	0.050	0.560	-0.801	1.161	0.929	-0.433	0.453

(1) not indicated due to limited interpretability

(2) z-transformed to a mean of zero and a standard deviation of one; original mean and standard deviation were *M* = 4.481 and *SD* = 2.443

(3) z-transformed to a mean of zero and a standard deviation of one; original mean and standard deviation were *M* = 3.511 and *SD* = 1.691

(4) z-transformed to a mean of zero and a standard deviation of one; original mean and standard deviation were *M* = 6.608 and *SD* = 3.444

(5) z-transformed to a mean of zero and a standard deviation of one; original mean and standard deviation were *M* = 40.012 and *SD* = 23.681

Table S5 Results of GLMM which investigates the effect of value on performance in the no-delay trials. (Estimates, together with standard errors (SE), confidence limits (Cl), and significance tests (P))

Term	Estimate	SE	Cl (lower)	Cl (upper)	P
Intercept	-2.353	1.318	-6.689	-0.141	(1)
Value (mixed value)	3.669	1.169	1.881	7.920	0.002
Session (2)	0.048	0.413	-0.814	0.893	0.907

(1) not indicated due to limited interpretability

(2) z-transformed to a mean of zero and a standard deviation of one; original mean and standard deviation were *M* = 3.511 and *SD* = 1.691

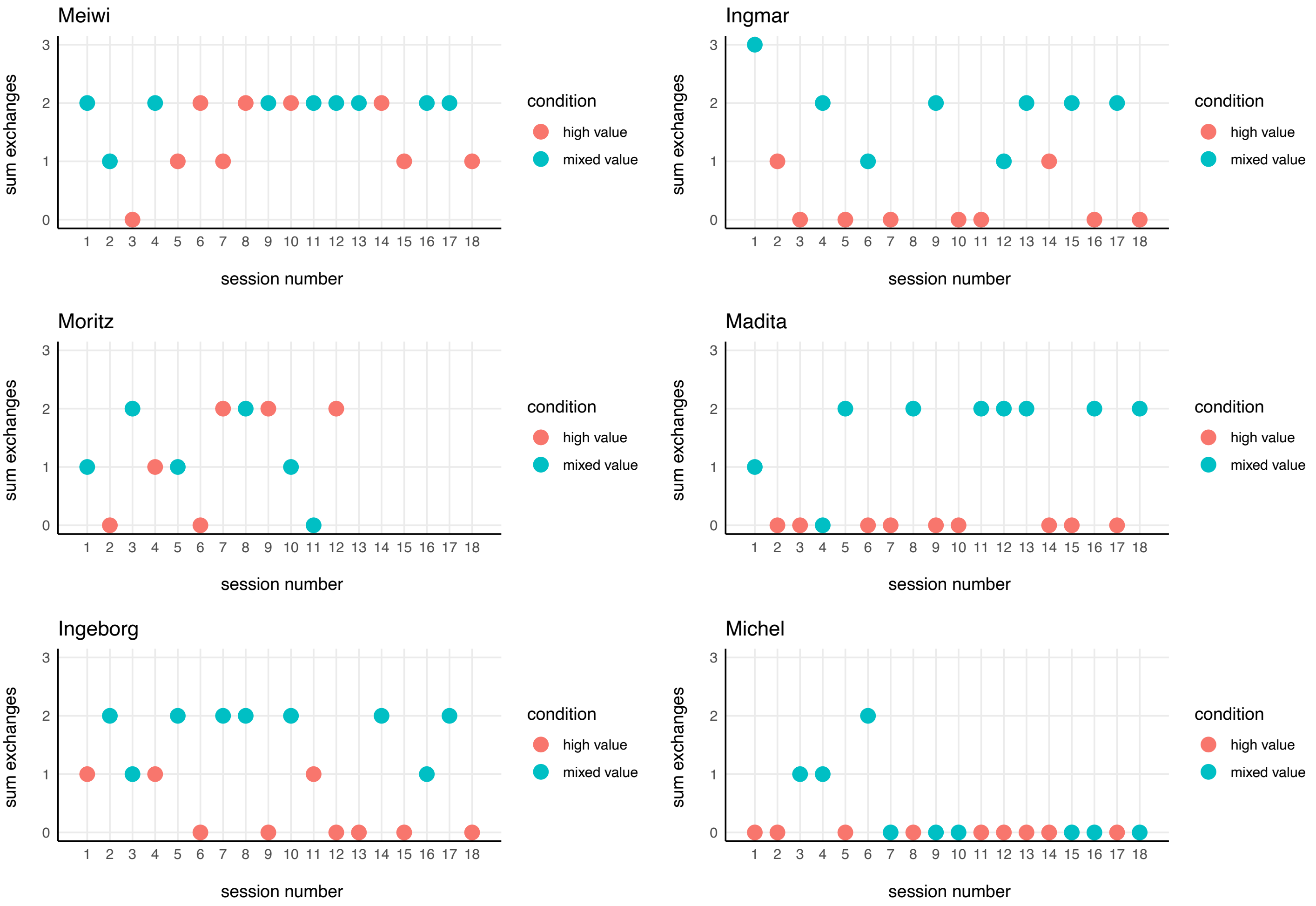

Figure S5 Performance in no-delay trials prior to each test session in the mixed and high value condition per subject. Before each test session, subjects received two *no-delay* trials in the respective condition. Ingmar received four trials in his first session before we adjusted the procedure to two trials for all following sessions. The order of condition presentation was pseudo-randomized such that for each session number at least two individuals received high-value and mixed-value condition.

References

Addessi, E., Crescimbene, L., & Visalberghi, E.: Do capuchin monkeys (*Cebus apella*) use tokens as symbols?. P. R. Soc. B., *274*(1625), 2579–2585. https://doi.org/10.1098/rspb.2007.0726, 2007

Addessi, E., Bellagamba, F., Delfino, A., De Petrillo, F., Focaroli, V., Macchitella, L., Maggiorelli, V., Pace, B., Pecora, G., Rossi, S., Sbaffi, A., Tasselli, M. I., & Paglieri, F.: Waiting by mistake: Symbolic representation of rewards modulates intertemporal choice in capuchin monkeys, preschool children and adult humans. Cognition, 130(3), 428–441. https://doi.org/10.1016/j.cognition.2013.11.019, 2014.

Addessi, E., Mancini, A., Crescimbene, L., & Visalberghi, E.: How social context, token value, and time course affect token exchange in capuchin monkeys (*Cebus apella*). Int. J. Primatol., 32(1), 83–98. https://doi.org/10.1007/s10764-010-9440-4, 2011.

Addessi, E., & Rossi, S.: Tokens improve capuchin performance in the reverse–reward contingency task. P. R. Soc. B., *278*(1707), 849-854. https://doi.org/10.1098/rspb.2010.1602, 2011.

Amici, F., Aureli, F., & Call, J.: Fission-fusion dynamics, behavioral flexibility, and inhibitory control in primates. Curr. Biol., *18*(18), 1415–1419. https://doi.org/10.1016/j.cub.2008.08.020, 2008.

Auersperg, A. M. I., Laumer, I. B., & Bugnyar, T.: Goffin cockatoos wait for qualitative and quantitative gains but prefer 'better' to 'more'. Biol. Letters., *9*(3), 20121092. https://doi.org/10.1098/rsbl.2012.1092, 2013.

Beran, M. J., & Evans, T. A.: Language-trained chimpanzees (*Pan troglodytes*) delay gratification by choosing token exchange over immediate reward consumption. Am. J. Primatol., *74*(9), 864–870. https://doi.org/10.1002/ajp.22042, 2012.

Beran, M. J., & Parrish, A. E.: Non-human primate token use shows possibilities but also limitations for establishing a form of currency. Philos. T. R. Soc. B., *376*(1819), 20190675. https://doi.org/10.1098/rstb.2019.0675, 2021.

Beran, M. J., Rossettie, M. S., & Parrish, A. E.: Trading up: Chimpanzees (*Pan troglodytes*) show self-control through their exchange behavior. Anim. Cogn., *19*(1), 109–121. https://doi.org/10.1007/s10071-015-0916-7, 2016.

Boysen, S. T., & Berntson, G. G.: Responses to quantity: Perceptual versus cognitive mechanisms in chimpanzees (*Pan troglodytes*). J. Exp. Psychol. Anim. B., *21*(1), 82–86. https://doi.org/10.1037/0097-7403.21.1.82, 1995.

Boysen, S. T., Berntson, G. G., Hannan, M. B., & Cacioppo, J. T.: Quantity-based interference and symbolic representations in chimpanzees (*Pan troglodytes*). J. Exp. Psychol. Anim. B., *22*(1), 76–86. https://doi.org/10.1037/0097-7403.22.1.76, 1996.

Bugnyar, T., Wascher, C., & Dufour, V.: Carrion crows cannot overcome impulsive choice in a quantitative exchange task. Front. Psychol., *3*, 118. https://doi.org/10.3389/fpsyg.2012.00118, 2012.

Carlson, S. M., Davis, A. C., & Leach, J. G.: Less is more: Executive function and symbolic representation in preschool children. Psychol. Sci., *16*(8), 609–616. https://doi.org/10.1111/j.1467-9280.2005.01583.x, 2005.

De Petrillo, F., Gori, E., Micucci, A., Ponsi, G., Paglieri, F., & Addessi, E.: When is it worth waiting for? Food quantity, but not food quality, affects delay tolerance in tufted capuchin monkeys. Anim. Cogn., *18*(5). https://doi.org/10.1007/s10071-015-0869-x, 2015.

Drapier, M., Chauvin, C., Dufour, V., Uhlrich, P., & Thierry, B.: Food-exchange with humans in brown capuchin monkeys. Primates, *46*(4), 241–248. https://doi.org/10.1007/s10329-005-0132-1, 2005.

Dufour, V., Pelé, M., Sterck, E. H. M., & Thierry, B.: Chimpanzee (*Pan troglodytes*) anticipation of food return: Coping with waiting time in an exchange task. J. Comp. Psychol., *121*(2), 145–155. https://doi.org/10.1037/0735-7036.121.2.145, 2007.

Evans, T. A., Beran, M. J., Paglieri, F., & Addessi, E.: Delaying gratification for food and tokens in capuchin monkeys (*Cebus apella*) and chimpanzees (*Pan troglodytes*): When quantity is salient, symbolic stimuli do not improve performance. Anim. Cogn., *15*(4), 539–548. https://doi.org/10.1007/s10071-012-0482-1, 2012.

Gazes, R. P., Billas, A. R., & Schmitt, V.: Impact of stimulus format and reward value on quantity discrimination in capuchin and squirrel monkeys. Learn. Behav., *46*(1), 89-100. https://doi.org/10.3758/s13420-017-0295-9, 2018.

Genty, E., Palmier, C., & Roeder, J.-J.: Learning to suppress responses to the larger of two rewards in two species of lemurs, *Eulemur fulvus* and *E. macaco*. Anim. Behav., *67*(5), 925–932. https://doi.org/10.1016/j.anbehav.2003.09.007, 2004.

Genty, E., & Roeder, J.-J.: Transfer of self-control in black (*Eulemur macaco*) and brown (*Eulemur fulvus*) lemurs: Choice of a less preferred food item under a reverse-reward contingency. J. Comp. Psychol., *121*(4), 354. https://doi.org/10.1037/0735-7036.121.4.354, 2007.

Grace, R. C., Sargisson, R. J., & White, K. G.: Evidence for a magnitude effect in temporal discounting with pigeons. J. Exp. Psychol. Anim. B., *38*(1), 102–108. https://doi.org/10.1037/a0026345, 2012.

Green, L., & Myerson, J.: A discounting framework for choice with delayed and probabilistic rewards. Psychol. Bull., *130*(5), 769 –792. https://doi.org/10.1037/0033-2909.130.5.769, 2004.

Green, L., Myerson, J., Holt, D. D., Slevin, J. R., & Estle, S. J.: Discounting of delayed food rewards in pigeons and rats: Is there a magnitude effect? J. Exp. Anal. Behav., *81*(1), 39–50. https://doi.org/10.1901/jeab.2004.81-39, 2004.

Green, L., Myerson, J., & McFadden, E.: Rate of temporal discounting decreases with amount of reward. Mem. Cognition., *25*(5), 715–723. https://doi.org/10.3758/BF03211314, 1997.

Green, L., Myerson, J., & Ostaszewski, P.: Amount of reward has opposite effects on the discounting of delayed and probabilistic outcomes. J. Exp. Psychol. Learn., *25*(2), 418–427. https://doi.org/10.1037/0278-7393.25.2.418, 1999.

Hillemann, F., Bugnyar, T., Kotrschal, K., & Wascher, C. A. F.: Waiting for better, not for more: Corvids respond to quality in two delay maintenance tasks. Anim. Behav., *90*, 1–10. https://doi.org/10.1016/j.anbehav.2014.01.007, 2014.

Judge, P. G., & Essler, J. L.: Capuchin monkeys exercise self-control by choosing token exchange over an immediate reward. Int. J. Comp. Psychol., *26*(4). https://doi.org/10.46867/ijcp.2013.26.04.05, 2013.

Kirby, K. N.: Bidding on the future: Evidence against normative discounting of delayed rewards. J. Exp. Psychol. Gen., *126*(1), 54–70. https://doi.org/10.1037/0096-3445.126.1.54, 1997.

Kirby, K. N., & Maraković, N. N.: Delay-discounting probabilistic rewards: Rates decrease as amounts increase. Psychon. B. Rev., *3*(1), 100–104. https://doi.org/10.3758/BF03210748, 1996.

Koepke, A. E., Gray, S. L., & Pepperberg, I. M.: Delayed gratification: A grey parrot (*Psittacus erithacus*) will wait for a better reward. J. Comp. Psychol., *129*(4), 339–346. https://doi.org/10.1037/a0039553, 2015.

Kralik, J. D.: Inhibitory control and response selection in problem solving: How cotton-top tamarins (*Saguinus oedipus*) overcome a bias for selecting the larger quantity of food. J. Comp. Psychol., *119*(1), 78–89. https://doi.org/10.1037/0735-7036.119.1.78, 2005.

Labuschagne, L. G., Cox, T.-J., Brown, K., & Scarf, D.: Too cool? Symbolic but not iconic stimuli impair 4-year-old children's performance on the delay-of-gratification choice paradigm. Behav. Process., *135*, 36–39. https://doi.org/10.1016/j.beproc.2016.11.014, 2017.

Leonardi, R. J., Vick, S.-J., & Dufour, V.: Waiting for more: The performance of domestic dogs (*Canis familiaris*) on exchange tasks. Anim. Cogn., *15*(1), 107–120. https://doi.org/10.1007/s10071-011-0437-y, 2012.

Miller, R., Frohnwieser, A., Schiestl, M., McCoy, D. E., Gray, R. D., Taylor, A. H., & Clayton, N. S.: Delayed gratification in New Caledonian crows and young children: Influence of reward type and visibility. Anim. Cogn., *23*(1). https://doi.org/10.1007/s10071-019-01317-7, 2020.

Orduña, V., Valencia-Torres, L., Cruz, G., & Bouzas, A.: Sensitivity to delay is affected by magnitude of reinforcement in rats. Behav. Process., *98*, 18–24. https://doi.org/10.1016/j.beproc.2013.04.011, 2013.

Pelé, M., Dufour, V., Micheletta, J., & Thierry, B.: Long-tailed macaques display unexpected waiting abilities in exchange tasks. Anim. Cogn., *13*(2), 263–271. https://doi.org/10.1007/s10071-009-0264-6, 2010.

Pepperberg, I. M., & Rosenberger, V. A.: Delayed gratification: A grey parrot (*Psittacus erithacus*) will wait for more tokens. J. Comp. Psychol., https://doi.org/10.1037/com0000306, 2022.

Rachlin, H.: On the tautology of the matching law. J. Exp. Anal. Behav., *15*(2), 249–251. https://doi.org/10.1901/jeab.1971.15-249, 1971.

Ramseyer, A., Pelé, M., Dufour, V., Chauvin, C., & Thierry, B.: Accepting loss: The temporal limits of reciprocity in brown capuchin monkeys. P. R. Soc. B., *273*(1583), 179–184. https://doi.org/10.1098/rspb.2005.3300, 2006.

Richards, J. B., Mitchell, S. H., de Wit, H., & Seiden, L. S.: Determination of discount functions in rats with an adjusting-amount procedure. J. Exp. Anal. Behav., *67*(3), 353–366. https://doi.org/10.1901/jeab.1997.67-353, 1997.

Rosati, A. G., Stevens, J. R., & Hauser, M. D.: The effect of handling time on temporal discounting in two New World primates. Anim. Behav., *71*(6), 1379–1387. https://doi.org/10.1016/j.anbehav.2005.11.012, 2006.

Rumbaugh, D. M., King, J. E., Beran, M. J., Washburn, D. A., & Gould, K. L.: A salience theory of learning and behavior: With perspectives on neurobiology and cognition. Int. J. Primatol., *28*(5), 973–996. https://doi.org/10.1007/s10764-007-9179-8, 2007.

Schmitt, V., & Fischer, J.: Representational format determines numerical competence in monkeys. Nat. Commun., *2*(1), 257. https://doi.org/10.1038/ncomms1262, 2011.

Stevens, J. R., & Mühlhoff, N.: Intertemporal choice in lemurs. Behav. Process., *89*(2), 121–127. https://doi.org/10.1016/j.beproc.2011.10.002, 2012.

Stevens, J. R., Rosati, A. G., Ross, K. R., & Hauser, M. D.: Will travel for food: spatial discounting in two new world monkeys. Curr. Biol., *15*(20), 1855-1860. https://doi.org/10.1016/j.cub.2005.09.016, 2005.
